# Practical COVID-19 Prevention Training for Obstetrics and Gynecology Residents Based on the Conceive–Design–Implement–Operate Framework

**DOI:** 10.3389/fpubh.2022.808084

**Published:** 2022-03-03

**Authors:** Xiaoxue Wang, Yangzi Zhou, Zixuan Song, Yuting Wang, Xueting Chen, Dandan Zhang

**Affiliations:** ^1^Department of Health Management, Shengjing Hospital of China Medical University, Shenyang, China; ^2^Department of Obstetrics and Gynecology, Shengjing Hospital of China Medical University, Shenyang, China; ^3^Medical Issue Mediation Office, Shengjing Hospital of China Medical University, Shenyang, China

**Keywords:** COVID-19, CDIO model, engineering education model, infectious disease protection, practical skills training, resident standardized training, working pressure, professional identity

## Abstract

**Background:**

The spread of COVID-19 poses a challenge for obstetrics and gynecology (O&G) residents. In order to improve the theoretical knowledge and practical skills of residents in epidemic prevention and control, reduce work pressure and improve professional skills, effective and sound training models are required to improve the protection of O&G residents from COVID-19.

**Method:**

A total of 38 standardized training O&G residents working in Shengjing Hospital of China Medical University in March 2020 was selected. They were randomly divided into intervention and control groups. The control group underwent a protection theory exposition according to the traditional training method, while the intervention group adopted a conceive–design–implement–operate (CDIO) mode, arranged training courses in combination with the O&G specialty, and completed four modules of CDIO. After the training, the theoretical knowledge and practical operation were assessed, and the work stress and occupational identity scales were assessed. The assessment results and scores of the two groups of residents were analyzed.

**Results:**

Compared with the scores of the residents in the control group, the theoretical and technical scores of the residents in the intervention group significantly improved (*P* < 0.05). In the evaluation of organizational management, workload, interpersonal relationship, and doctor–patient relationship pressure, the scores of the intervention group were lower than those of the control group, with a statistical difference (*P* < 0.05). For the intervention group, the job stress and professional identity evaluation scores were significantly higher than those of the control group (*P* < 0.05).

**Conclusion:**

The CDIO model can effectively enhance the theoretical knowledge and practical skills of O&G residents in COVID-19 epidemic prevention protocols to reduce work pressure and improve professional identity. In addition, it provides new ideas, methods, and approaches for future clinical practice training.

## Introduction

COVID-19 has been declared an “International Public Health Emergency” by the World Health Organization (WHO) because the virus is extremely contagious and has spread globally. The COVID-19 pandemic has profoundly changed the academic and clinical learning environment of obstetrics and gynecology (O&G) in many ways. The rapid development of virus variants affects patients, doctors, and medical students (learners). This calls for unprecedented collaboration and rapid and profound readjustment, practically every day.

Residents undertake a lot of responsibility in medical clinical work, including ward duty, medical records, operation assistance, outpatient care, and emergency support. Medical institutions have conducted regular training on infection management and COVID-19 protocols for residents in the initial stage of the epidemic (1). However, owing to the lack of clinical experience and the limitations of responding ability (2), minimal theoretical teaching cannot sufficiently induce an awareness of the importance and key points of epidemic prevention and control. In addition, because of the specialty of O&G, the diagnosis and treatment of pregnant women during the epidemic remained the same (3). Therefore, the prevention and control of the epidemic in the outpatient clinic and ward of O&G residents are extremely important. Furthermore, it is vital to conduct standardized training, implement standard protective protocols, establish training programs for infectious disease protection, and perform strict, efficient, timely, and professional practical training for O&G residents. Traditional (teacher-centered) teaching methods were adopted in early COVID-19 obstetric training sessions. After lectures on COVID-19-related knowledge and demonstrations on personal protective equipment (PPE) operation, the residents passively received theoretical instruction. When the residents were sent to the epidemic area to actually work, they only mechanically completed relevant operations in the infected environment, and their subjective initiatives failed to be stimulated in a short time. After the training of resident physicians, the level of mastery of O&G knowledge and the combination of clinical practice in the infectious environment could not meet the needs of the cultivation and development of clinical practice ability. Effective and sound training models are required to improve professional practical skills and epidemic response ability.

O&G residents have to face high intensity and high risk of work pressure, long-term high load, irregular work and rest time, and need to deal with sudden disease changes at any time. They are prone to tension, anxiety and other emotions, because the body is constantly in a state of high stress. With China's medical reform, women have increasingly higher requirements for the quality of medical services, and higher expectations for disease treatment. The lack of effective communication skills and the tension between doctors and patients caused great mental pressure to O&G residents. At the same time, residents also have to face with pressure from career development, interpersonal relationship, career interest and external environmental factors.

Professional identity refers to the psychological identity of one's own career and the ability to positively perceive and evaluate related aspects of the career. The higher the professional identity, the higher the work enthusiasm and efficiency. Medical staff with low professional identity are prone to work pressure, job burnout or resignation thoughts. O&G residents are new to clinical work, busy work and low salary which inevitably lead to doubts about their career. Improving the professional identity of medical staff can promote the work efficiency and physical and mental health of medical staff.

The theory of conceive–design–implement–operate (CDIO) is based on “learning by doing”. It is the latest achievement in international engineering education reform^1^. It considers product research and development using the whole life cycle of the product operation as the carrier and realizes the organic combination of initiative, realization, and courses, to improve students' comprehensive quality, such as their engineering system abilities, teamwork abilities, personal abilities, and basic engineering knowledge (4). This model emphasizes “student-centred” teaching, in which the students participate in the conception, design, implementation, and operation of the project and turn the theoretical knowledge into a tool to solve problems (5). The CDIO model has been gradually applied in medical vocational education systems (6). Teachers use CDIO in various resident trainings, such as physical examinations, auxiliary examinations, information integration, diagnoses and identification, treatment plans, and education, by constructing simulation scenes and presenting cases. Clinical practice confirmed that this model had positive significance in training innovative and comprehensive medical talent.

This study explored the application effect of the CDIO model in the practical training of O&G residents during the COVID-19 pandemic, and evaluates the psychological status of O&G residents using the Job Stressor Scale and Professional Identity.

## Materials and Methods

### Subjects

A total of 38 residents who receive standardized training in the O&G base of Shengjing Hospital of China Medical University in March 2020 was selected. Based on the actual situation, the residents were randomly divided into two groups for training depending on the ward as a unit. There were 18 and 20 residents in the control and intervention groups, respectively. There were no statistically significant differences in gender, age, educational structure, and training duration between the groups (P > 0.05), as shown in Table 1.

**Table 1:** Characteristics of residents in the two groups.

**Items**	**Control group** **(*n* = 18)**	**Intervention group** **(*n* = 20)**	**Statistics**	***P***
**Age**	24.63 ± 1.48	25.11 ± 1.12	*t* = −1.134	0.264
**Gender [** ***N*** **, (** ***n*** **%)]**			χ^2^ = 0.257	0.612
Male	1	2		
Female	17	18		
**Education [** ***N*** **, (n%)]**			χ^2^ = 0.368	0.544
Undergraduate	15	18		
Postgraduate	3	2		
**Training duration [** ***N*** **, (n%)]**			χ^2^ = 0.326	0.572
6 months	12	13		
18 months	3	4		
30 months	3	3		

### Traditional Training Methods

A specialist from the Department of Infection Prevention and Control provided an intensive exposition on COVID-19 protection theory, including the following: ① grading standards of self-protection; ② correct wearing of PPE; ③ diagnoses and treatment procedures for patients with fever in wards, outpatient, and emergency; ④ diagnoses and treatment procedures for O&G operations during the epidemic; ⑤ prevention, control, and referral procedures for suspected or confirmed patients. The specialist provided a step-by-step demonstration of the basic use of PPE to the residents. After the demonstration, the residents practiced independently, and an assessment was conducted.

### CDIO Professional Training

The intervention group adopted the CDIO professional training model. Training courses were arranged according to the characteristics of O&G, and the theoretical content was written by specialists from the Department of Infection Prevention and Control. The specialists uniformly studied professional CDIO training courses online prior to the training and clarified the CDIO training model. According to the “Novel Coronavirus Prevention and Control Technical Guide (First Edition) in Medical Institutions” and “The Guidelines on the Scope of Use of Common Medical Protective Articles in the Prevention and Control of *novel coronavirus*-infected *pneumonia* (Trial)” issued by the National Health Commission in China, the preparation of lesson plans and operation and implementation plans were uniformly conducted.

The specific content of the epidemic prevention practice programme for the residents in the intervention group is shown in Table 2. For example, the specific training process for the simulation scenario “emergency room reception of suspected COVID-19 cases in labor” is as follows.

**Table 2:** Epidemic prevention practice training programme for residents in the intervention group using the CDIO model.

**Steps**	**Items**	***Teaching* contents**	***Teaching methods***	***Teaching* goals**
Conceive, C	*Construction of professional thinking*	1. Introduction of cases of suspected COVID-19 patients in obstetric emergency rooms prior to the training, leading to problems related to emergency protection. 2. Residents consulted references and discussed in groups. 3. Define a series of protection procedures and plans based on the case.	*Case*-based *teaching CBL teaching*	1. Cultivate specialized thinking of infectious diseases. 2. Improve the literature retrieval ability of residents. 3. Enhance the basic professional knowledge of residents.
Design, D	Project module design	1. Protective articles preparation: personal and patient protective items, doctor–patient environmental isolation, and clinical family resettlement. 2. Medical cooperation in the emergency room: cooperation during obstetric examinations, disinfection of related examination items, establishment of medical aseptic barriers, and shift and management of staff. 3. Emergency-ward transport process: isolation and disinfection during transport, protection of transport personnel and items, and ward reception plan.	Participatory teaching	1. Improve residents' professional practical ability in obstetrics and gynecology. 2. Establish awareness of infection prevention and control in emergency reception and transportation. 3. Enhance the overall prevention and control concept of medical cooperation.
Implement, I	Project practice	1. The group completed the scenario drill according to the design. 2. Coordinate with each other and be corrected under the guidance of the trainer.	Simulation training Role-playing	1. Train residents in communication and coordination skills; 2. Enhance residents' team awareness.
Operate, O	Results show	1. The team completed the emergency flow chart of cases and reported it 2. Trained teachers to complete comments and summaries	Multimedia teaching	1. Improve the ability of residents to find and solve problems. 2. Exercise residents' ability to explore knowledge in practice.

Step 1, Conceive (C): Two days prior to the training, the specialists selected practical cases in line with the current pandemic, and proposed specialized protection problems for emergency O&G were sent out via WeChat. For example, a 31-year-old female primipara was admitted with the chief complaint of “vaginal bleeding for 10 h and fever for 1 h”. The patient developed a large amount of vaginal fluid 10 h earlier without induction, abdominal pain, and vaginal bleeding and felt fever 1 h earlier with a self-measured body temperature of 37.2 °C. The patient was rushed into the emergency room by an emergency ambulance. History: Her husband went out for dinner a week ago, accompanied by unknown people, not excluding contacts from other provinces. An emergency physical examination revealed the following: body temperature, 37.3 °C; heart rate, 104 beats/min; respiratory rate, 28 breaths/min; and blood pressure, 130/80 mmHg. An obstetric examination revealed the following: uterine height, 41 cm; abdominal circumference, 105 cm; fetal heart, 164 beats/min; the cervix was soft and dilated in the center. A pH test was conducted, and the paper turned blue. The fetal heart monitoring revealed a reactive type with no evident contractions. Certain problems related to emergency obstetric protection were proposed, as shown in Table 2. The residents were required to consult relevant literature and discuss them in groups.

Step 2, Design (D): After listening to the discussion and analysis of each group, the specialists designed work items for obstetric emergency epidemic prevention and control as follows. ① Isolation mechanisms for doctors attending emergencies (preparation of medical supplies for clinical reception, isolation method when receiving patients, handover and cooperation with an emergency ambulance, isolation of the clinical history collection, and transportation protection when completing auxiliary examination); ② cooperation and protection of emergency nurses during obstetric examination (disinfection and isolation of obstetric examination instruments, protection during obstetric examination, treatment of medical waste, disinfection of examination bed, and therapeutic department); and ③ protection scheme of patients' family and inspectors in relevant auxiliary departments. In addition to professional PPE, emphasis was placed on the cultivation of the residents' and nursing team's abilities to deal with problems, communication skills, critical thinking, and comprehensive knowledge application.

Step 3, Implement (I): The team completed the scenario simulation according to the design. Each group comprised four residents, who functioned as the emergency physician, emergency room attending physician, emergency room nurse, and ward attending physician, respectively. Each resident performed situational simulation drills according to his/her role setting, focusing on PPE wearing and taking off, isolation management techniques, and protective management of patient handover tasks. During the simulation process, if there was any question or practice bottleneck, the residents communicated with the specialists.

Step 4, Operate (O): Finally, each group summarized the entire process of case reception, treatment, and transfer in the form of flow charts or mind maps. The specialists commented on and modified the advantages and disadvantages of the plan and provided a detailed rectification in line with practical scenarios.

### Training Effect Evaluation

After the training, unified COVID-19 prevention and control papers were used for testing, and theoretical scores were compared for the two groups. The full score of the paper was 100 points, including 80 points for single-choice questions and 20 points for four short-answer questions. Concurrently, the evaluation standard of the PPE protective operation provided by the hospital was used as the scoring basis to evaluate the operation of residents in the two groups.

The Chinese Physician's Job Stressor Scale (CPJS) (7) was used to evaluate the residents. The scale has seven dimensions, including organizational management, career interest, workload, career development, interpersonal relationship, doctor–patient relationship, and external environment, which were divided into 31 items. Each item was divided into four answers: strongly disagree, somewhat disagree, somewhat agree, and strongly agree, and they were assigned points of 1–4, respectively. The higher the score, the greater the work pressure. The Cronbach's α coefficient for the scale was 0.881.

The professional identity of the residents was scored using a scale published by Li (8). This scale comprised 13 items regarding professional identity. These items were assessed on a five-point Likert scale, ranging from 1 (strongly disagree) to 5 (strongly agree) (Table 3). The total scores ranged from 13 to 65, with high scores indicating high professional identity among the residents. Scores of 13–17 indicated a low level of professional identity, 18–33 indicated a medium level of professional identity, and ≥ 34 indicated a high level of professional identity.

**Table 3:** Professional identity assessment scale in the two groups.

	**Control group (*****n*** **=** **18)**					**Intervention group (*****n*** **=** **20)**						
**Items**	**Strongly disagree** ***n* (%)**	**Strongly disagree** ***n* (%)**	**Neutral** ***n* (%)**	**Somewhat agree** ***n* (%)**	**Strongly agree** ***n* (%)**	**Strongly disagree** ***n* (%)**	**Strongly disagree** ***n* (%)**	**Neutral** ***n* (%)**	**Somewhat agree** ***n* (%)**	**Strongly agree** ***n* (%)**	**χ^2^/t**	***P***
1. When referring to my profession, I usually say “we” rather than “they”.	2 (11.11%)	6 (33.33%)	8 (44.44%)	1 (5.56%)	1 (5.56%)	0 (0.00%)	1 (5.00%)	2 (10.00%)	12 (60.00%)	5 (25.00%)	102.21	<0.001
2. I consider my success the success of healthcare workers.	2 (11.11%)	5 (27.78%)	9 (50.00%)	0 (0.00%)	2 (11.11%)	0 (0.00%)	0 (0.00%)	2 (10.00%)	9 (45.00%)	9 (45.00%)	106.18	<0.001
3. I care very much about other people's views on my career.	2 (11.11%)	4 (22.22%)	10 (55.56%)	2 (11.11%)	0 (0.00%)	0 (0.00%)	1 (5.00%)	3 (15.00%)	9 (45.00%)	7 (35.00%)	99.97	<0.001
4. The praise my profession receives from people = the praise I receive.	1 (5.56%)	4 (22.22%)	11 (61.11%)	1 (5.56%)	1 (5.56%)	0 (0.00%)	2 (10.00%)	1 (5.00%)	10 (50.00%)	7 (35.00%)	102.97	<0.001
5. If there are criticisms about my career from the media, I will feel ashamed and embarrassed.	1 (5.56%)	5 (27.78%)	10 (55.56%)	1 (5.56%)	1 (5.56%)	0 (0.00%)	0 (0.00%)	2 (10.00%)	11 (55.00%)	7 (35.00%)	105.40	<0.001
6. My job is important/I believe in the importance of my job.	0 (0.00%)	7 (38.89%)	9 (50.00%)	1 (5.56%)	1 (5.56%)	0 (0.00%)	0 (0.00%)	0 (0.00%)	11 (55.00%)	9 (45.00%)	112.33	<0.001
7. I am confident in my ability to work.	2 (11.11%)	1 (5.56%)	10 (55.56%)	3 (1.67%)	2 (11.11%)	0 (0.00%)	1 (5.00%)	1 (5.00%)	10 (50.00%)	8 (40.00%)	97.55	<0.001
8. My job will improve patients' conditions.	1 (5.56%)	3 (16.67%)	9 (50.00%)	4 (22.22%)	1 (5.56%)	0 (0.00%)	0 (0.00%)	0 (0.00%)	12 (60.00%)	8 (40.00%)	103.58	<0.001
9. My job is meaningful.	3 (16.67%)	3 (16.67%)	9 (50.00%)	1 (5.56%)	2 (11.11%)	0 (0.00%)	0 (0.00%)	1 (5.00%)	9 (45.00%)	10 (50.00%)	105.36	<0.001
10. I have the necessary qualifications and skills for my job.	4 (22.22%)	4 (22.22%)	7 (38.89%)	2 (11.11%)	1 (5.56%)	0 (0.00%)	0 (0.00%)	2 (10.00%)	11 (55.00%)	7 (35.00%)	102.59	<0.001
11. I understand the responsibilities and requirements of my work.	3 (1.67%)	5 (27.78%)	5 (27.78%)	4 (22.22%)	1 (5.56%)	0 (0.00%)	1 (5.00%)	2 (10.00%)	9 (45.00%)	8 (40.00%)	95.00	<0.001
12. Healthcare work suits me.	2 (11.11%)	6 (33.33%)	5 (27.78%)	3 (16.67%)	2 (11.11%)	0 (0.00%)	1 (5.00%)	1 (5.00%)	12 (60.00%)	6 (30.00%)	96.40	<0.001
13. I know my role.	3 (16.67%)	5 (27.78%)	5 (27.78%)	4 (22.22%)	1 (5.56%)	0 (0.00%)	0 (0.00%)	0 (0.00%)	10 (50.00%)	10 (50.00%)	104.10	<0.001
Total Score	32.55 ± 6.54					84.00 ± 7.13					23.10	<0.001

### Statistical Analysis

Data were entered into Epidata (version 3.1) and analyzed using SPSS (version 21.0). The counting data were expressed in frequencies and percentages, and the measurement data were expressed as mean ± standard deviation ($\bar{x}$± *s*). Prior to the statistical analysis, the comparison data were normally distributed. Subsequently, count data were compared using χ^2^ tests, and measurement data were compared using the independent sample *t*-test for the two groups. A *p*-value < 0.05 was considered statistically significant.

## Results

### Theoretical and Skill Assessment Scores for the Two Groups

After the training, the score of protection theory for the intervention group was 92.76 ± 4.38, which was significantly higher than that of the control group (*t* = 5.861, *P* < 0.001). In the skill assessment, the average score of residents in the intervention group was 87.33 ± 4.90, and that in the control group was 80.84 ± 2.65. The score of residents in the intervention group was significantly higher than that in the control group (*t* = 4.996, *P* < 0.001).

### Job Stress Scores for the Two Groups

A total of 38 questionnaires were issued, and 38 valid questionnaires were recovered, with an effective recovery rate of 100%. According to the different dimensions of the questionnaires, the assessment results of job stress and professional identity of the residents in the two groups were statistically analyzed. Compared with the control group, the evaluation of organizational management, workload, interpersonal relationships, and doctor–patient relationships significantly improved in the intervention group (P < 0.05). The results are listed in Table 4.

**Table 4:** The Chinese Physician's Job Stressor Scale (CPJS) in the two groups ($\bar{x}$ ± s).

**Items**	**Control group** **(*n* = 18)**	**Intervention group** **(*n* = 20)**	**t**	***P***
Organizational management	2.72 ± 0.61	2.24 ± 0.69	−2.262	0.030
Career interest	2.19 ± 0.65	2.21 ± 0.68	0.089	0.927
Workload	3.55 ± 0.74	3.08 ± 0.58	−2.190	0.035
Career development	2.66 ± 0.59	2.71 ± 0.55	0.271	0.790
Interpersonal relationship	2.24 ± 0.64	1.64 ± 0.69	−2.769	0.009
Doctor–patient relationship	3.26 ± 0.64	2.74 ± 0.75	−2.286	0.028
External environment	3.58 ± 0.62	3.61 ± 0.56	0.157	0.876

### Professional Identity for the Two Groups

After different training modes, the residents of the two groups were evaluated using a professional identity scale. The intervention group scored higher on average than the control group (P < 0.01) and exhibited a higher sense of professional identity, as shown in Table 3.

## Discussion

### The Remarkable Effect of Epidemic Prevention Training in O&G Using the CDIO Model

Traditional infection prevention and control training employs a one-way infusion teaching method, focusing on explanations and demonstrations; however, it cannot effectively combine infection prevention and control and clinical thinking systematically. As a new method of education and training, CDIO (9) enables students to actively combine theory with clinical practice and is conducive for the training of lifelong knowledge acquisition abilities. O&G is a highly practical field and a first-line clinical high-risk discipline during COVID-19 and other infectious disease outbreaks (10). To improve the training level and clinical emergency ability of residents, a scientific teaching model is required. Using the CDIO model, training and guidance are conducted by instructors through group collaboration to solve clinical problems of O&G in practice. Here, after applying different teaching methods to complete the training, the performance of residents in the intervention group was significantly better than that in the control group in terms of theoretical and practical assessments (*P* < 0.05), suggesting that the CDIO teaching model can improve the training effect for residents. In addition, the integration of the CDIO model into the epidemic prevention and control training in O&G departments can effectively divide and integrate O&G departments with infection prevention and control, outpatient, and emergency departments; ward and medical care departments; and related auxiliary departments and expand the depth of the teaching system based on a multi-layer teaching platform, such that all levels connect and complement each other.

### CDIO Model Is Beneficial in Relieving the Occupational Stress of O&G Residents During the Pandemic

The sudden outbreak of the pandemic was a majorly stressful event for residents who recently commenced clinical work. Although medical practitioners were aware of the severity of COVID-19, certain residents felt anxious at the beginning of the spread of the epidemic because the future was unclear (11). In particular, for the O&G residents at the front line of the clinical work, owing to the rigid demand in obstetrics, it was difficult to artificially control the number of patients and the intensity of work. In addition, they faced the pressure of dual medical security for pregnant women and newborns. Concurrently, at the clinical front line, they experienced severe outcomes due to viral infections and panicked at the possibility of iatrogenic transmission to relatives and friends (12). Traditional training methods adopt centralized teaching, resulting in a scattered knowledge of epidemic prevention and control, lack of overall understanding of professional protection, and relatively slow improvement in comprehensive protection ability. In addition, owing to the lack of scene fidelity, practical training projects for process development, and corresponding practical training methods, the practical training was disconnected from the real front-line protection work. This hinders residents from objectively viewing the current pandemic, calmly and properly dealing with problems and difficulties generated at the clinical front-line, thereby hindering the correct prevention and control education of Non-medical personnel around them (13). After adopting the CDIO model, teachers were trained to set up simulation scenarios and guide residents to follow the CDIO process to improve the evaluation, diagnosis, planning, measures, and evaluation of medical procedures through field cases. In addition to mastering knowledge points, resident doctors in training can clarify the context of knowledge according to simulated cases, systematically combine professional knowledge with epidemic prevention, and enhance their ability to flexibly deal with problems in practice. Therefore, in this study, the job stress of residents in the intervention group was lower than that in the control group in terms of organizational management, workload, interpersonal relationship, and doctor–patient relationship, and the difference was statistically significant (P < 0.05), particularly in the element of interpersonal relationship. However, for the career interest, career development, and external environmental factors, the effect of the CDIO teaching model was not significant. This may be related to the occupational risk factors of O&G and the uncertain trend of COVID-19, which requires further study.

### CDIO Model Training Is Beneficial in Increasing the Professional Identity of O&G Residents

Each section of the CDIO model training requires close cooperation among residents, nurses, and medical auxiliary personnel (14, 15). Therefore, different roles need to be simulated in the group to negotiate and solve problems. This is helpful for residents to master the overall medical process, stimulate potential, and cultivate teamwork and communication skills. In addition, by role-playing in clinical cases, the field situation can be simulated to discover and solve problems in practice and improve the comprehensive ability of disease assessment, critical thinking, problem-solving, and professional identity. Here, the evaluation scores of residents in the intervention group for job stress and professional identity were significantly higher than those in the control group (P < 0.05), which indicated that the CDIO training model had an improvement effect on the professional identity of residents.

### Limitations

The main limitation of this study is the small sample size. Single-center research is one reason and the number of O&G residents included is small each year, which is related to the size of the hospital. These make the statistical results of this study uncertain. Of course, on the other hand, this CDIO is carried out for the first time in the education of O&G residents, which is a predictive test in a small population. Future research needs to expand the study population, preferably by perfecting multi-center studies, with the expectation of widespread application.

## Conclusion

In conclusion, the CDIO model can effectively enhance the ability of O&G residents to integrate theoretical knowledge and practical skills of COVID-19 prevention. It enhances the training effect, reduces the work pressure of residents, and improves their professional identity. When suddenly faced with COVID-19 and other similar infectious diseases, increase in clinical workload, and high risk of infection in medical care, the CDIO model provides manpower guarantee for specialized treatment and epidemic prevention for O&G patients. Finally, it provides new ideas, methods, and approaches for clinical skills and practical training in the future.

## Data Availability Statement

The original contributions presented in the study are included in the article/supplementary material, further inquiries can be directed to the corresponding author.

## Author Contributions

XW and DZ designed the study and drafted the manuscript. YZ, ZS, and YW designed the statistical analysis plan. XC and DZ reviewed the manuscript. All authors take responsibility for the appropriateness of the content.

## Funding

This research was supported by internal funding from Shengjing Hospital, China Medical University (SJ-M0133), and 345 Talent Project of Shengjing Hospital of China Medical University (No. M0946).

## Conflict of Interest

The authors declare that the research was conducted in the absence of any commercial or financial relationships that could be construed as a potential conflict of interest.

## Publisher's Note

All claims expressed in this article are solely those of the authors and do not necessarily represent those of their affiliated organizations, or those of the publisher, the editors and the reviewers. Any product that may be evaluated in this article, or claim that may be made by its manufacturer, is not guaranteed or endorsed by the publisher.

## Footnotes

^1^Available at: http://www.cdio.org/.
